# Small Non-Coding RNA Profiling in Plasma Extracellular Vesicles of Bladder Cancer Patients by Next-Generation Sequencing: Expression Levels of miR-126-3p and piR-5936 Increase with Higher Histologic Grades

**DOI:** 10.3390/cancers12061507

**Published:** 2020-06-09

**Authors:** Alexandru A. Sabo, Giovanni Birolo, Alessio Naccarati, Mihnea P. Dragomir, Serena Aneli, Alessandra Allione, Marco Oderda, Marco Allasia, Paolo Gontero, Carlotta Sacerdote, Paolo Vineis, Giuseppe Matullo, Barbara Pardini

**Affiliations:** 1 Klinikum Stuttgart, Olgahospital, Zentrum für Kinder, Jugend und Frauenmedizin, Pediatrics 2 (General and Special Pediatrics), 70174 Stuttgart, Germany; saboalexandru@gmail.com; 2 Department of Pediatrics, Marie Curie Emergency Clinical Hospital for Children, 041434 Bucharest, Romania; 3 Department of Medical Sciences, University of Turin, 10126 Turin, Italy; giovanni.birolo@unito.it (G.B.); serena.aneli@unito.it (S.A.); alessandra.allione@unito.it (A.A.); giuseppe.matullo@unito.it (G.M.); 4 Italian Institute for Genomic Medicine (IIGM) 10060 Candiolo, Italy; alessio.naccarati@iigm.it (A.N.); p.vineis@imperial.ac.uk (P.V.); 5 Candiolo Cancer Institute, FPO-IRCCS, 10060 Candiolo, Italy; 6 Department of Surgery, Fundeni Clinical Hospital, Carol Davila University of Medicine and Pharmacy, 022328 Bucharest, Romania; mihnea.p.dragomir@gmail.com; 7 Department of Surgical Sciences, University of Turin and Città della Salute e della Scienza, 10126 Turin, Italy; marco.oderda83@gmail.com (M.O.); marc.allasia@gmail.com (M.A.); paolo.gontero@unito.it (P.G.); 8 Center for Cancer Prevention (CPO-Piemonte), 10126 Turin, Italy; carlotta.sacerdote@cpo.it; 9 MRC-HPA Centre for Environment and Health, School of Public Health, Imperial College London, London W2 1PG, UK

**Keywords:** bladder cancer, extracellular vesicles, next-generation sequencing, liquid biopsy, small non-coding RNA profiling, microRNAs, piRNAs, non-invasive biomarkers

## Abstract

Bladder cancer (BC) is the tenth most frequent cancer worldwide. Due to the need for recurrent cystoscopies and the lack of non-invasive biomarkers, BC is associated with a high management burden. In this respect, small non-coding RNAs (sncRNAs) have been investigated in urine as possible biomarkers for BC, but in plasma their potential has not yet been defined. The expression levels of sncRNAs contained in plasma extracellular vesicles (EVs) from 47 men with BC and 46 healthy controls were assessed by next-generation sequencing. The sncRNA profiles were compared with urinary profiles from the same subjects. miR-4508 resulted downregulated in plasma EVs of muscle-invasive BC patients, compared to controls (adj-*p* = 0.04). In World Health Organization (WHO) grade 3 (G3) BC, miR-126-3p was upregulated both in plasma EVs and urine, when compared to controls (for both, adj-*p* < 0.05). Interestingly, two sncRNAs were associated with the risk class: miR-4508 with a downward trend going from controls to high risk BC, and piR-hsa-5936 with an upward trend (adj-*p* = 0.04 and adj-*p* = 0.05, respectively). Additionally, BC cases with low expression of miR-185-5p and miR-106a-5p or high expression of miR-10b-5p showed shorter survival (adj-*p* = 0.0013, adj-*p* = 0.039 and adj-*p* = 0.047, respectively). SncRNAs from plasma EVs could be diagnostic biomarkers for BC, especially in advanced grade.

## 1. Introduction

Bladder cancer (BC) ranks as the tenth most frequent diagnosed human cancer worldwide, with 549,393 new cases and 199,922 related deaths being reported in 2018, according to the GLOBOCAN statistics [1].

BC has a marked male predominance, with a 3:1 male-to-female ratio [2]. BC incidence peaks at an older age (5th to 7th decade) and is strongly connected to the habit of smoking. Occupational and environmental carcinogens are additional contributing risk factors. BC is more prevalent in countries with a high human development index [2,3]. Moreover, in some world regions, like Northern and sub-Saharan Africa, higher prevalence is attributed to the *Schistosoma haematobium* infection [4].

Based on tissue infiltration, BC is usually divided into two separate entities: non-muscle invasive bladder cancer (NMIBC), accounting for 70% of the cases, and muscle invasive bladder cancer (MIBC) [5]. Characteristics for NMIBC are high progression and recurrence rates that require repeated cystoscopic follow-up [6]. 

Unlike colorectal or breast cancers, there are no currently available screening programs for BC. Usual referral symptoms in BC patients are either macroscopic hematuria or urinary tract symptoms together with microscopic hematuria [4]. Urine cytology, which implies the microscopic examination of voided urine, is one of the current non-invasive diagnostic tools, albeit with limited utility, because of the relatively low sensitivity, especially in low-grade tumors (16%) [7]. Therefore, the current gold standard for diagnosis is represented by cystoscopy and tissue biopsy, an invasive procedure that causes major patient discomfort [8] and potential adverse effects [6]. The replacement of the invasive and costly procedure of cystoscopy is one of the main objectives in biomarker development for BC [9].

Researchers have been active in seeking new molecules that could act as biomarkers in either diagnosis or follow-ups. One such class of molecules are the small non-coding RNAs (sncRNAs). They are transcripts, less than 200 nucleotides in length, which are not translated into proteins. The most investigated sncRNAs are microRNAs (miRNAs), 19–22 nucleotide long molecules, which serve predominantly as post-transcriptional modulators [10]. Among other sncRNAs, Piwi-interacting RNAs (piRNAs) are slightly longer molecules (26–31 nucleotides) implicated in transposon silencing and transcriptional regulation [11]. 

SncRNAs are expressed in tissues, but they can also be isolated from body fluids, where they can be found either as cell-free circulating molecules (cfRNA) or packed in extracellular vesicles (EVs), such as exosomes [12]. In recent years, the interest on sncRNAs has increased since they have been found dysregulated in cancer and they have been identified as potential biomarkers for cancer diagnosis and monitoring [13].

For BC, due to the direct contact to the tumor and ease of sampling, urine is the most investigated body fluid, with promising results reported by our group [14]. Other investigators searched for BC biomarkers also in other readily available body fluids, such as blood-derived plasma or serum. In this sense, most of the studies investigated cfRNA profiles but there are suggestions to start focusing on circulating EVs and their cargos [15]. However, the results are still inconclusive and, in general, the available studies did not investigate the potentiality of these biomarkers in distinguishing among subtypes of BC [16]. 

**Figure 1:**
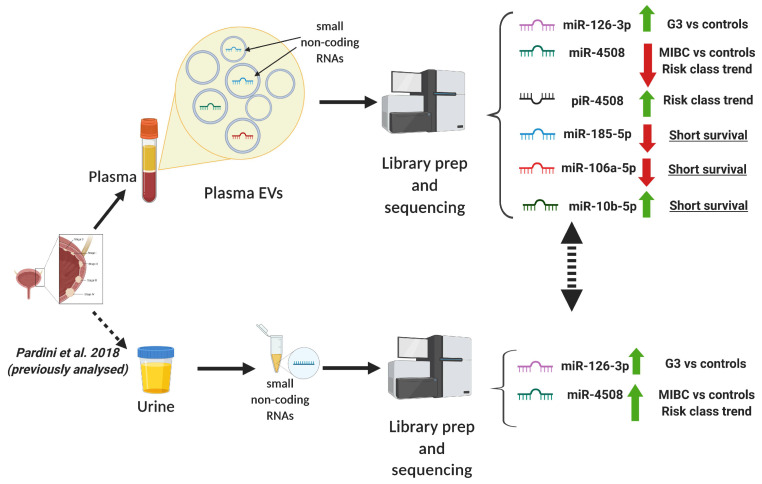
Schematic overview of the experimental approach and main results obtained.

In the present study, we investigated sncRNA profiles by next-generation sequencing (NGS) in plasma-derived EVs from BC patients and healthy controls with the aim of finding potential non-invasive biomarkers for the diagnosis and prognosis of BC. Additionally, we compared the current results with the sncRNA expression data measured in urine from the same patients previously reported by our group [14] (Figure 1).

## 2. Results

### 2.1. Sample Characteristics

Out of the 93 subjects analyzed, 47 were cases with BC (8 MIBC and 39 NMIBC) and 46 were age-matched controls. When using the 1973 World Health Organization (WHO) classification of histologic grading (G), out of 39 NMIBC, 12, 16, and 11 resulted G1, G2 and G3, respectively. Applying the updated 2004/2016 WHO classification, 17 NMIBC patients were classified as low-grade papillary urothelial carcinoma (LG) while 22 NMIBC and 8 MIBC were high-grade papillary urothelial carcinoma (HG). The characteristics of the study population are summarized in Table 1.

### 2.2. Sequencing Results

For the aforementioned samples, four library pools (24-indexed samples per pool) were prepared for deep sequencing. An average of 11 million reads per sample were generated, ranging from 2.4 to 33 million reads. Raw reads were trimmed for adaptor sequence; reads with length less than 14 nucleotides were discarded. An average of 1.2 million reads per sample (from 0.04 to 7.10 million) were correctly mapped to mature sncRNA sequences collected from miRBase (release 22), piRBase (version 1.0) and Ensembl (release 91). Considering all samples, an average of 759 unique sncRNAs (of which 566 miRNAs) were identified and associated with at least one read, ranging from 358 to 1242 unique sncRNAs (from 270 to 968 unique miRNAs). After the mapping step, we created a count matrix composed by 93 samples and 2408 sncRNAs having at least one read in one sample. Depending on the selected model (see material and methods section), the number of sncRNAs tested for differential expression ranged from 474 to 517. A summary of the results is presented in Table S1.

### 2.3. Differentially expressed sncRNAs in Extracellular Vesicles 

We tested several models to assess whether there were differentially expressed sncRNAs in EVs of BC patients compared to healthy controls. All models were adjusted by batch (library pool), age, and smoking status. Hereby, we only report associations that are significant after accounting for the testing of multiple sncRNAs (False Discovery Rate (FDR)). 

The first comparison was between all cancer cases and controls, yielding no significant association. Then, we stratified the cases according to three criteria: tumor type (NMIBC vs MIBC), and two histologic gradings (G1 and G2 vs. G3, or LG vs. HG). For each stratification, we compared the resulting subgroups and each of them against the controls, totaling nine comparisons. 

**Figure 2:**
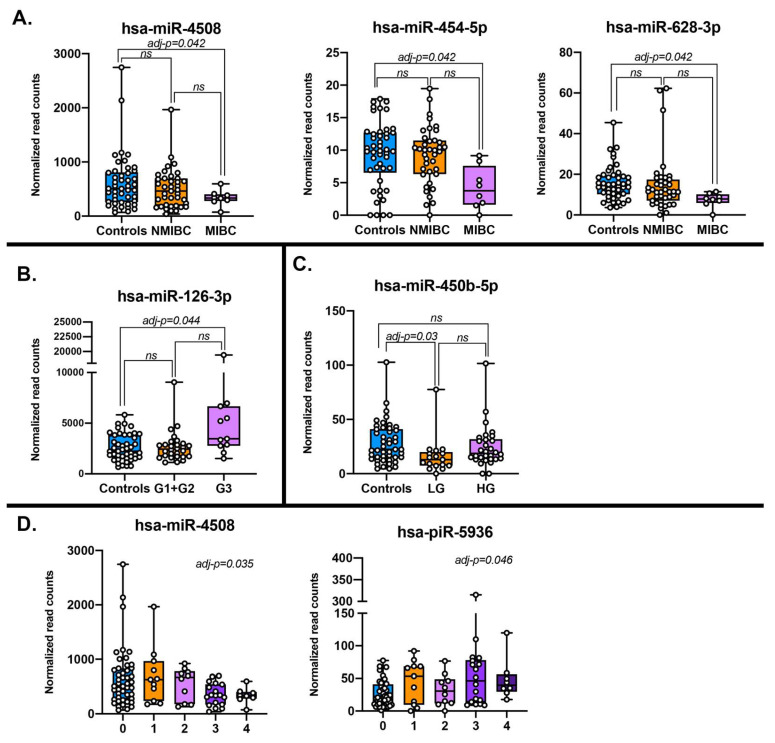
Normalized read counts of differentially expressed sncRNAs across various comparisons according to different stratification of the study population: (**A**) Bladder cancer (BC) diagnosis (MIBC = muscle invasive bladder cancer; NMIBC = non-muscle invasive bladder cancer; and controls); (**B**) WHO 1973 histological grade (G3, G1+G2 and controls); (**C**) WHO 2004 histological grade (HG, LG and controls); and (**D**) risk class (0 = controls, 1 = low risk, 2 = intermediate risk, 3 = high risk, 4 = MIBC).

For the tumor type, the only comparison yielding differentially expressed sncRNAs was MIBC versus controls. Specifically, four miRNAs were downregulated in MIBC: miR-4508 (adj-*p* = 0.04), miR-454-5p (adj-*p* = 0.04), miR-628-3p (adj-*p* = 0.04), and miR-3140-3p (adj-*p* = 0.01). However, results should be taken cautiously since only miR-4508 had high read counts (653 on average) while the other three miRNAs were much less abundant (less than 20) with miR-3140-3p having 3.2 reads on average (not shown) (Figure 2A, Table 2). 

When considering WHO 1973 histological grading, miR-126-3p resulted upregulated (adj-*p* = 0.04) in NMIBC G3 when compared to controls (Figure 2B, Table 2). No differentially expressed sncRNAs were found comparing NMIBC G1+G2 against G3 or controls.

By stratifying cases according to the more recent WHO 2004 histological grading, the only significant dysregulated sncRNA was miR-450b-5p, which was significantly downregulated in LG cases with respect to controls (adj-*p* = 0.03; Figure 2C, Table 2). The same results were obtained either including or not MIBC in the HG class (Figure 2C reported the plot including MIBC).

Finally, the associations with the risk class were also tested and two differentially expressed sncRNAs were found associated: miR-4508 (adj-*p* = 0.04) with a downward trend going from controls to high risk and MIBC (class 4) and piR-hsa-5936 (adj-*p* < 0.05) with an upward trend (Figure 2D, Table 2).

### 2.4. Replication of the Differentially Expressed sncRNAs in Urine of the Same Subjects

We checked if the altered sncRNAs in plasma EVs were also differentially expressed in urine from the same patients (as already reported in [14]). miR-3140-3p and miR-454-5p were not detected in urine samples while miR-450b-5p and piR-hsa-5936 were not significantly differentially expressed. On the other hand, miR-628-3p and miR-4508 were significantly upregulated in urine, in contrast to plasma where they were downregulated. Only miR-126-3p was significantly upregulated in G3 with respect to controls in both plasma EVs and urine, with lower expression (~100 reads in average) but higher fold change in urine (adj-*p* = 1.04 × 10^−5^). All the results are reported in Table 2.

### 2.5. Comparison with miRNA Profiles in Primary Tissues from TCGA

We also checked whether the differentially expressed miRNAs in plasma EVs observed in our study had similar expression patterns in tissue samples from MIBC patients (the only available) from The Cancer Genome Atlas (TCGA) project. We compared only tumor paired with normal tissues from the same individuals. In this case, miR-3140-3p, miR-4508, and piR-hsa-5936 were not expressed or quantified, while miR-628-3p, miR-450b-5p, and miR-126-3p were detected but not differentially expressed in tissues. miR-454-5p was significantly downregulated in cancer tissues. In plasma EVs, miR-454-5p was also downregulated in MIBC against controls (Table 2).

### 2.6. Functional Implications of Dysregulated miRNAs

We retrieved the validated target genes for the significantly differentially expressed miRNAs (namely, miR-450b, miR-126-3p, and miR-4508) and performed gene ontology (GO) and pathway enrichment analyses using the miRWalk 3.0 database and EnrichR separately for each of them.

miR-126-3p has 4 validated target genes (*GRIN2B, FOXO3, ADGRE5,* and *CXCL12*). Several terms were enriched after statistical adjustment for multiple tests including biological processes involving the regulation of death of neuronal cells (GO Biological Processes: “positive regulation of neuron death” (GO:1901216), “negative regulation of dendritic cell apoptotic process” (GO:2000669), “regulation of dendritic cell apoptotic process” (GO:2000668), etc.; (Table S2A) and the inflammatory response (GO Molecular Functions: “CXCR chemokine receptor binding (GO:0045236)”, “chemokine activity (GO:0008009)”, “cytokine receptor binding (GO:0005126)”; KEGG: “Chemokine signaling pathway”, “NF-κ B signaling pathway”, “Intestinal immune network for IgA production”; Table S2A).

miR-4508 has 12 validated target genes (*ASB6, ABL1, YES1, LEPROTL1, VAV3, IRAK3, U2AF2, PEX26, RGS6, HOOK3, KIAA0754, CAPN15*). Enrichment analyses linked these genes to several GO Biological Process terms including those mediating the immune system response (“Fc-gamma receptor signaling pathway” (GO:0038094), “Fc-gamma receptor signaling pathway involved in phagocytosis” (GO:0038096), “Fc receptor mediated stimulatory signaling pathway” (GO:0002431)) and signal transduction (“peptidyl-tyrosine autophosphorylation” (GO:0038083), “transmembrane receptor protein tyrosine kinase signaling pathway” (GO:0007169), “cellular protein modification process” (GO:0006464), etc.; for all adj-*p* < 0.005; Table S2B). Searching in the GO Molecular Functions database, target genes of miR-4508 were enriched in several terms involved in the modification of proteins (“protein phosphorylated amino acid binding” (GO:0045309), “phosphotyrosine residue binding” (GO:0001784), and “protein tyrosine kinase activity” (GO:0004713)).

Finally, miR-450b-5p has 5 validated target genes (*SR8SIA3, CNEP1R1, VMA21, POGK*, and *RAET1E*) which are linked to several pathways and molecular processes, among the others “natural killer cell mediated cytotoxicity” (KEGG), “natural killer cell lectin-like receptor binding” (GO Molecular Function, GO:0046703), “alpha-N-acetylneuraminate alpha-2,8-sialyltransferase activity” (GO Molecular Function, GO:0003828) and “sialyltransferase activity” (GO Molecular Function, GO:0008373) (Table S2C).

### 2.7. Predictivity of sncRNAs in BC Diagnosis

We ascertained the predictivity of 661 selected sncRNAs from plasma EVs for the following binary classification problems: cases versus controls and pairwise comparisons by tumor type (MIBC, NMIBC and controls) and WHO 1973 histological grade (G3, G1+G2, and controls) (Figure S1; Table S3). The highest median balanced accuracy (across a ten-fold cross-validation) in distinguishing MIBC from NMIBC was 0.63 and was obtained by the AdaBoost method testing all sncRNA expression levels together with age and smoke covariates. The same methods and the same classification were applied using the altered sncRNAs detected in urine from the same patients. sncRNAs from urine were consistently at least as predictive as those from plasma EVs and often they performed better (Figure S1).

### 2.8. Bladder Cancer Prognosis and Survival

Finally, we performed univariate and multivariate Cox regressions and log-rank test to compare Kaplan–Meier curves on cancer specific mortality survival (CSM), recurrence/progression-free survival (RPS), and event-free survival (EFS). All patients for whom the cause of death was uncertain were removed. In this way, we retained a total of 45 cases. In total, five individuals died for BC, 15 recurred, one had a progression, and one had both a recurrence event and progressed to MIBC.

After multiple test correction (FDR), significant results were found only for the CSM survival analyses. In particular, cases with a low expression of miR-185-5p or miR-106a-5p have a higher risk of death with respect to other cases (log-rank test adj-*p* = 0.0013 and log-rank test adj-*p* = 0.039, respectively; Figure S2A and B). Conversely, cases with a high expression of miR-10b-5p had shorter survival than other samples (log-rank test adj-*p* = 0.047; Figure S2C).

miR-185-5p, whose expression shows the strongest association with CSM, targets the 3′-UTRs of 111 validated genes. A Gene Set Enrichment Analysis (GSEA) resulted in interesting GO Biological Processes (Table S2D), such as “mitotic DNA integrity checkpoint” (adj-*p* = 0.001), “positive regulation of cell cycle arrest” (adj-*p* = 0.001) and “mitotic DNA damage checkpoint” (adj-*p* = 0.001), thus connecting this miRNAs with a putative function of a tumor suppressor through the regulation of cell proliferation. Similarly, WikiPathways 2019 Human database from EnrichR reported the enrichment of miR-185-5p targets in the pathway “Regulation of Microtubule Cytoskeleton WP2038” (adj-*p* = 0.003, Table S2D).

## 3. Discussion

We investigated by high throughput sequencing technology the sncRNA profiles in plasma derived EVs in all stages of BC and matched controls, with the aim to find non-invasive biomarkers that could be a valid alternative to cystoscopy. We observed a downregulation of miR-4508 in MIBC cases compared to controls and a downward trend of expression for miR-4508 and an upward trend of expression for piR-hsa-5936 with an increased BC risk class. Finally, we found that the expression levels of three sncRNAs (miR-185-5p, miR-106a-5p, and miR-10b-5p) are associated with survival in BC (Figure 1).

Biomarker studies only recently started to focus on the content of EVs. In the past, the main objects of study in body fluids were free-circulating mRNAs and miRNAs especially as a diagnostic tool for BC [9]. The new challenge is to analyze the role of other sncRNAs, especially those carried in EVs, as prognostic and predictive markers [9]. In this respect, the present study focused on profiling by NGS sncRNAs contained in EVs from plasma. EVs mediate the communication between cancer cells and the microenvironment supporting the development of the tumor but also preparing pre-metastatic niche via systemic circulation to distant sites [9,13]. In BC research, urine is the most investigated body fluid because of the direct contact with the tumor tissue. However, studying circulating sncRNAs in other body fluids, especially in EVs, could generate better biomarkers for monitoring BC patients and for their follow up since EVs are secreted by tumor cells in the blood stream to exert an effect in distant cells [9].

As recently reviewed, there is a broad range of studies focusing on the use of urinary miRNAs as biomarkers for BC [9,15,17]. Notably, we found only four studies on urine-derived EVs in association with BC, which reported among the others miR-21-5p [18,19,20], miR-375 [21], miR-200-family [19,20], and miR-146 [21,22] as potential diagnostic biomarkers. Few researchers investigated circulating blood-derived products in BC. For example, Usuba et al. recently proposed a panel of 7 freely-circulating miRNAs able to discriminate BC from healthy controls and other malignancies with an AUC of 0.98 (sensitivity 95%, specificity 87%) [23]. Data on circulating biomarkers in BC has been summarized by Khetrapal et al. [24], but to the best of our knowledge, no studies were conducted so far on the role of sncRNAs contained in EVs derived from plasma/serum neither as diagnostic nor prognostic biomarkers.

According to the available literature, this is also the first study assessing circulating sncRNAs derived from plasma EVs by NGS for BC. The use of the NGS is an additional advantage: most of the studies used microarray techniques, which are prone to bias due to dependency on reference genomes and possible errors in cross-hybridization. On the other hand, with its ability to detect changes at the nucleotide level, NGS offers more reliable and reproducible results [25].

GO and pathway enrichment analyses on the validated target genes of the dysregulated sncRNAs, show several cancer-related processes enriched, especially for the validated targets of miR-126-3p. This miRNA has been previously linked to BC [26] and others cancers [27], as well as to angiogenesis [28,29]. Grimolizzi et al., for example, reported miR-126-3p to induce cell proliferation and angiogenesis in non-tumorigenic cells, but also acting at the same time as a tumor suppressor and metastasis inhibitor by targeting *IRS1, EGFL7, Crk, SLC7A513* [30,31]. In non-small-cell lung cancer, the expression levels of miR-126-3p in plasma decreased with the progressive cancer grade, while increased in serum exosomes [30]. Since a preferential accumulation of this miRNA in plasma/serum exosomes has been reported, we could hypothesize that miR-126-3p secretion is an active tumor process, aiming to stimulate angiogenesis in nearby and distant healthy tissues and facilitating metastasis. Additional functional studies will be necessary to confirm this role in BC together with further validation of this signal in an independent cohort. miR-450b-5p has been indicated as an active repressor of stemness in colorectal cancer [32] and has been involved in the p53 signaling pathway [33]. This miRNA has also been studied in association with progression of several solid tumors including, lung adenocarcinoma [34], rectal cancer [35], and prostate cancer [36]. Interestingly, miR-450b-5p has been associated with ageing being detected in serum-derived exosomes from old rats [37]. Other two sncRNAs, miR-4508 and piR-hsa-5936, found dysregulated in plasma EVs by us, have never been associated to BC. Additional mechanistic studies are warranted to confirm their functions.

Concerning the CSM survival of BC patients, miR-185-5p, miR-106a-5p, and miR-10b-5p expression levels seem to be relevant for the prognosis of BC patients. In our study, low expression levels of miR-185-5p and miR-106a-5p or the high expression of miR-10b-5p were associated with a short survival. miR-185-5p has been already reported in the literature as a potential cancer biomarker for melanoma [38], prostate [39], and bladder cancer [14,40]. However, the expression rates of this miRNA are not consistent across studies, thus raising some issues about the possibility to clarify its complex molecular involvement in tumor progression, also considering the heterogeneity of tissues and cancer types. As reported in [15,38] and suggested by the target gene enrichments, this miRNA acts as a regulator of cellular proliferation. Therefore, it is plausible a scenario where its expression levels are high in correspondence to the urine [14,40] (i.e., to induce cellular proliferation) but this effect is not visible in plasma.

miR-106a-5p has also been described as a potential suppressor of proliferation, migration, and invasion of BC cells [41], supporting our finding that low-expression are associated with worse prognosis. On the other hand, the function of miR-10b-5p is less clear, several studies have validated its role as a metastasis promoting factor, with aberrant expression across different cancer types [42]. In BC, the significance of miR-10b-5p remains controversial. On one hand, its overexpression has been linked to increased migration, invasion, and metastasis in cell lines and animal models [43]. On the other hand, it was found downregulated in tumor tissues with respect to adjacent normal tissues, and had no significant effect on prognosis and survival [43,44]. However, the reduced number of deceased cases (due to the low mortality rate of BC) does not warrant further speculations on their mechanistic role in BC.

We are aware of some limitations of the present study. In fact, we have included 47 BC patients and 46 age-matched controls which constitute a relatively small sample size. It is not easy to collect a large study population with multiple tissues and appropriate sampling procedures, and with the exception of large studies such as TCGA, the other available studies reported similarly sized BC populations. For the same reason it was not possible to perform any validation on an independent cohort. BC is almost three times more common in men than in women; therefore, male patients are relatively “easier” to collect. In this respect, a study only on men may be biased, but at the same time may reduce the variability in the analysis of a relatively small sample size. Moreover, it has been demonstrated that women usually are diagnosed with BC in more advanced stages and they have shorter survival times than men. Some factors associated with a history of cystitis may contribute to or explain the poorer outcome, regardless of treatment and after adjustment for a range of other prognostic variables [45]. For the future studies, it could be therefore especially useful to expand the analyses on larger cohorts that will include also women with BC.

Moreover, the overall normalized read counts of the detected miRNAs were relatively low. This was somewhat expected since it is quite well-known that the amount of isolated RNA carried by EVs is low [46,47] and, in particular, there is a selection of the miRNAs cargo transported by EVs. For sncRNA species other than miRNAs, less information is currently available. Finally, although we hypothesized potential destinations of EVs secreted from BC tumor cells, the target genes of these sncRNAs were not experimentally determined. More in-depth and mechanistic studies are necessary to determine the target organs of these differentially expressed circulating sncRNAs.

## 4. Materials and Methods

### 4.1. Patients

The study population consisted of 93 men enrolled in the Turin Bladder Cancer Study (TBCS) [48,49]. Out of them, 47 were diagnosed with BC and 46 were age-matched controls. Among BC, 8 were diagnosed as MIBC and 39 as NMIBC. Patients were all newly diagnosed, histologically confirmed cases of BC registered at two Urology Departments of A.O.U. Città della Salute e della Scienza, in Turin (Italy). Controls were males recruited randomly from patients treated at the same urology departments for non-neoplastic disease (prostatic hyperplasia, cystitis, and others) or from patients treated at the medical and surgical departments for hernias, vasculopathies, diabetes, heart failure, asthma, or other benign diseases. Patients with cancer, liver, or renal diseases, and smoking-related conditions were excluded. All the patients signed a written consent to participate in the study according to the Helsinki Declaration.

The study was approved by the Interhospital Ethical Board of San Giovanni Battista/C.T.O./C.R.F./Maria Adelaide hospitals (Turin, Italy) and the Institutional Review Boards of the Italian Institute for Genomic Medicine (IIGM). From all study participants, urine and plasma samples were collected. miRNA expression levels measured in urine have been previously reported in [14] (Figure 1).

### 4.2. Histological Grading of Bladder Cancers

Regarding the histologic grading for BC, the 1973, 2004 and 2016 WHO classifications were considered. The 1973 WHO classification uses cellular and architectural atypia to subgroup NMIBC patients into three grades: G1, G2, and G3. The 2004/2016 WHO classifications were introduced because of reported unclear pathological situations of tumors having criteria of belonging to two classes (G1/2 and G2/3). As a consequence, the G1-3 grading was changed into papillary urothelial neoplasm of low malignant potential (PUNLMP), non-invasive low-grade papillary urothelial carcinoma (LG), and high-grade papillary urothelial carcinoma (HG), respectively [50].

### 4.3. Risk Group Stratification

The BC risk classification was also evaluated according to European Urology Association (EUA) guidelines [5]. Therefore, BC patients were classified into 4 risk classes—low, intermediate, and high risk, and MIBC based on multiple criteria. Classification criteria included macroscopic (size, aspect, number of tumors) and microscopic characteristics (invasiveness—T grade, histologic grade, lympho-vascular invasion).

### 4.4. Plasma Separation and Extracellular Vesicles Precipitation

For all subjects, human plasma samples were obtained from 5–8 mL of blood centrifuged for 10 min at 1000 rpm. Plasma aliquots (about 200–300 μL each) were then stored at −80 °C until use. EVs were isolated from 200 μL of plasma using the ExoQuick exosome precipitation solution (System Biosciences, Mountain View, CA, USA) according to the manufacturer’s instructions and [51]. Briefly, plasma was mixed with 50.4 μL of ExoQuick solution and refrigerated at 4 °C overnight (at least 12 h). The mixture was further centrifuged at 1500 g for 30 min. The EVs pellet was dissolved in 200 μL of nuclease-free water; RNA was extracted immediately from this solution.

### 4.5. RNA Extraction and Quality Control

Total RNA from plasma EVs was extracted with the miRNeasy plasma/serum mini kit (Qiagen, Hilden, Germany) using the QiaCube extractor (Qiagen, Germany). RNA quality and quantity were verified according to MIQE guidelines [52]. For all samples, RNA concentration was quantified by Invitrogen Qubit^®^ 4 Fluorometer with Qubit^®^ microRNA Assay Kit (Invitrogen, Milan, Italy).

### 4.6. Library Preparation for Small RNA-Seq

Small RNA transcripts were converted into barcoded cDNA libraries. Library preparation was performed with the NEBNext Multiplex Small RNA Library Prep Set for Illumina (New England BioLabs Inc., USA). For each library, 6 μL of RNA (min 35 ng) were used in all the experimental procedures as starting material. Each library was prepared with a unique indexed primer so that the libraries could all be pooled into one sequencing lane. Multiplex adaptor ligations, reverse transcription primer hybridization, reverse transcription reaction, and PCR amplification were performed according to the protocol for library preparation (Protocol E7330, New England BioLabs Inc., Ipswich, MA, USA). After PCR amplification, the cDNA constructs were purified with the QIAQuick PCR Purification Kit (Qiagen, Germany) following the modifications suggested by the NEBNext Multiplex Small RNA Library Prep Protocol and loaded on the Bioanalyzer 2100 (Agilent Technologies, Milan, Italy) using the DNA High Sensitivity Kit (Agilent, Germany) according to the manufacturer’s protocol. Libraries were pooled together (24-plex) and further purified with a gel size selection.

A final Bioanalyzer 2100 run with the High Sensitivity DNA Kit (Agilent Technologies, Milan, Italy) that allows the analysis of DNA libraries regarding size, purity and concentration completed the workflow of library preparation. The obtained libraries were subjected to the Illumina sequencing pipeline, passing through clonal cluster generation on a single-read flow cell (Illumina Inc., San Diego, CA, USA) by bridge amplification on the cBot (TruSeq SR Cluster Kit v3-cBOT-HS, Illumina Inc.) and 50 cycles sequencing-by-synthesis on the HiSeq 2000 (Illumina Inc.) (in collaboration with EMBL, Gene core facility, Heidelberg, Germany).

### 4.7. Computational and Statistical Analyses

Raw reads adapter clipping was performed with the Cutadapt software (version 1.18) [53]. Reads longer than 14 nucleotides were mapped to a sncRNA reference with the bwa alignment software (version 0.7.17-r1188) [54], using the mem algorithm and a seed length of 10. Only alignments without mismatches or indels were considered and those with the highest quality were used to assign each read to a unique sncRNA. Thus, sncRNAs were quantified for each sample and then merged into a single count matrix, setting missing sncRNAs to zero. Differential expression analysis was performed with the DESeq2 Bioconductor’s package (version 1.22.2) [55]. For each model, samples with missing covariates were dropped and only sncRNAs, where at least 70% of the remaining samples had counts greater than 5, were tested. sncRNAs were considered significantly associated with a condition or a trend if their p-value, after adjustment for multiple testing by FDR, was below the 0.05 threshold.

miRNA target genes were retrieved by miRWalk 3.0 database [56]. EnrichR was used for GO and pathway enrichment analyses [57,58].

To confirm that our findings reflected the situation in cancer tissues, we downloaded miRNA quantifications measured in BC tissue samples and adjacent normal mucosa from the bladder cancer dataset (BLCA) of the TCGA project. We tested for differential expression between tumor and healthy tissue samples taken from the same individuals. Since only 19 individuals had both samples available, we tested only these subgroups of pairs with DESeq2 (as already done in [14]). Unfortunately, the arms of the mature miRNAs detected (-5p or -3p) were not differentiated or reported in TCGA quantification, thus we lack this piece of information.

To ascertain the predictivity of sncRNAs, we employed different machine learning methods from the Python module scikit-learn (version 21.3): logistic regression with lasso and elastic net penalty, random forest classifier, and AdaBoost classifier. For lasso and elastic net penalties we tested two values for the C parameter: 1 (the default) and 0.1. Since C is the inverse of the regularization strength, the 0.1 value yields classifiers that use less features. Performance was estimated by ten-fold cross-validation for ten different permutations of the samples in our dataset. We measured performance with balanced accuracy (from scikit-learn) since some classes had a very uneven number of samples (e.g., MIBC versus controls). We discarded sncRNAs that were not expressed in more than half of the samples. For the remaining species, expression levels were normalized across samples by dividing them by the 98-percentile of the expression of each sample, regularized by f(e) = log(e + 0.01). The regularized expression levels of each sncRNA were further standardized by computing their z-scores, which were used as features in the various models. Besides expression levels, the included features were age (in years) and smoking (as a one-hot-encoded categorical variable).

To explore the associations of sncRNAs expression with survival, recurrence and progression occurrence, we evaluated the CSM survival, RPS, and EFS on BC cases. Before conducting the analyses, we removed all patients for whom the cause of death was uncertain. CSM survival was evaluated as the time from the diagnosis to the date of death or the last follow-up. In the RPS analyses, we considered the time from the diagnosis to the first event of either recurrence or progression, whichever came first. In the EFS, we applied the same criteria of RPS also considering death events.

We performed univariate and multivariate Cox regression, and Kaplan-Meier curves with log-rank test using *coxph* and *survfit* functions in R (packages *survival* and *survminer*, R version 3.5.2 [59]). Multivariate analyses were adjusted for age, smoking status, risk class and sequencing library.

Kaplan–Meier curves and their statistical tests were performed splitting cases into two groups by their expression levels for each sncRNA. Two thresholds were selected, taking into account the proportion of events. For instance, in the CSM analysis, there were five events out of 45 cases, corresponding to 11%. Then, for each sncRNA, two comparisons were performed: the lowest 11% expressed cases versus the remaining 89% and the top 11% expressed cases against the remaining 89%.

## 5. Conclusions

In the present study, profiles of selected sncRNAs in plasma EVs were able to distinguish MIBC from NMIBC, although their accuracy is too low to be useful in practice. We think that sncRNAs from plasma EVs could have the potential to be used as predictive biomarkers but further studies in larger cohorts with more balanced classes are necessary (for example with a larger sample size of MIBC or also including women).

Although significant results regarding miRNAs as diagnostic tools have been proposed in both blood-derived and urine biospecimen separately, a higher accuracy might be achieved by combining measurements of different classes of ncRNAs from different body fluids. Indeed, some authors have already proposed diagnostic biomarker combinations of different RNA classes [60], with excellent results. Recent advances in this field are promising and await translation into clinical practice.

## Figures and Tables

**Table 1:** Demographic characteristics of patients and controls included in the study.

Covariates	Categories	Cases *n* = 47	Controls *n* = 46	*p*-Value
Age	Mean (Median)	65.0 (66.4)	64.3 (65.7)	0.91
	Range	47.7–73.7	46.4–74.5	
Smoking	Non-smoker	5	5	0.95
	Former smoker	26	24	
	Current smoker	16	17	
WHO 1973	G1	12		
	G2	16		
	G3	11		
WHO 2004/2016	HG **	30		
	LG	17		
Tumor type	NMIBC	39		
	MIBC	8		
Risk *	1	11		
	2	10		
	3	18		
	4 (MIBC)	8		

* Risk class stratification according to the EAU [6]. ** Including MIBC. HG: high grade; LG: low grade; NMIBC: non-muscle invasive bladder cancer; MIBC: muscle invasive bladder cancer.

**Table 2:** Summary of differentially expressed sncRNAs across the various comparisons.

sncRNAs	Source	Comparison	Base Mean	log2 FC	Nominal *p*-Value	Adjusted *p*-Value *
miR-126-3p	Plasma EVs	G3 vs CO	3571.00	0.83	8.46 × 10^−5^	4.43 × 10^−2^
miR-126-3p	Urine	G3 vs CO	101.00	2.41	2.11 × 10^−7^	1.63 × 10^−6^
miR-126	Tissue (TCGA)	Tumor vs Normal	5504.14	0.26	9.35 × 10^−2^	1.31 × 10^−1^
miR-3140-3p	Plasma EVs	MIBC vs CO	3.16	−3.46	2.05 × 10^−5^	1.06 × 10^−2^
miR-4508	Plasma EVs	MIBC vs CO	653.24	−1.23	1.62 × 10^−4^	4.16 × 10^−2^
miR-4508	Urine	MIBC vs CO	54.28	1.24	9.38 × 10^−3^	2.48 × 10^−2^
miR-4508	Plasma EVs	Risk class trend	542.91	−0.20	1.12 × 10^−4^	3.54 × 10^−2^
miR-4508	Urine	Risk class trend	231.79	0.32	1.98 × 10^−3^	6.40 × 10^−3^
miR-450b-5p	Plasma EVs	LG vs CO	26.10	−1.10	5.92 × 10^−5^	3.05 × 10^−2^
miR-450b-5p	Urine	LG vs CO	5.26	−0.07	8.96 × 10^−1^	1.00
miR-450b	Tissue (TCGA)	Tumor vs Normal	53.7	−0.59	3.87 × 10^−2^	5.85 × 10^−2^
miR-454-5p	Plasma EVs	MIBC vs CO	9.99	−1.36	3.22 × 10^−4^	4.16 × 10^−2^
miR-454	Tissue (TCGA)	Tumor vs Normal	26.37	−1.05	1.48 × 10^−7^	6.74 × 10^−7^
miR-628-3p	Plasma EVs	MIBC vs CO	16.58	−1.22	3.09 × 10^−4^	4.16 × 10^−2^
miR-628-3p	Urine	MIBC vs CO	11.27	2.32	7.84 × 10^−5^	3.76 × 10^−4^
miR-628	Tissue (TCGA)	Tumor vs Normal	31.68	0.23	4.18 × 10^−1^	4.87 × 10^−1^
piR-hsa-5936	Plasma EVs	Risk class trend	37.01	0.29	2.92 × 10^−4^	4.61 × 10^−2^
piR-hsa-5936	Urine	Risk class trend	540.58	−0.09	2.86 × 10^−1^	3.89 × 10^−1^

log2 FC: log2 Fold change; EVs: extracellular vesicles; * FDR significant results in bold. miRNAs in TCGA lack the specification of -5p or -3p arms. CO = controls; MIBC = muscle-invasive bladder cancer; LG = low grade; EVs = extracellular vesicles; TCGA = The Cancer Genome Atlas.

## Supplementary Materials

The following are available online at https://www.mdpi.com/2072-6694/12/6/1507/s1, Figure S1: Assessment of the predictivity of sncRNAs from plasma EVs and from urine using different machine learning methods (logistic regression with lasso and elastic net penalty, random forest classifier, and AdaBoost classifier) for some binary classification problems: cases versus controls and pairwise comparisons by tumor type (MIBC, NMIBC and controls) and WHO 1973 histological grade (G3, G1+G2 and controls). Balanced accuracy of 0.5 is the baseline level of a random classifier. In general, urine sncRNAs perform better than their counterparts in EVs; Figure S2: Kaplan–Meier CSM curves in BC patients stratified for the expression levels of: A) miR-185-5p; B) miR-106a-5p; and C) miR-10b-5p; Table S1. Summary of sequencing results; Table S2. Enrichment analyses for the validated target genes of significantly DE miRNAs using the miRWalk 3.0 database and EnrichR: A) miR-126-3p; B) miR-4508; and C) miR-450b. Only significant enrichments are shown; Table S3: Machine learning methods to evaluate the predictivity of sncRNAs in the distinction of bladder cancer: logistic regression with lasso and elastic net penalty, random forest classifier and AdaBoost classifier.
