# Supplementary material for: Small Non-Coding RNA Profiling in Plasma Extracellular Vesicles of Bladder Cancer Patients by Next-Generation Sequencing: Expression Levels of miR-126-3p and piR-5936 Increase with Higher Histologic Grades
Source: Cancers (Basel). 2020 Jun 9;12(6):1507. doi: 10.3390/cancers12061507 (PMC7352804; doi:10.3390/cancers12061507)
Supplement: Supplementary file 1 [file cancers-12-01507-s001.pdf]

Supplemental Materials: Small Non-Coding RNA Profiling in Plasma Extracellular Vesicles of Bladder Cancer Patients by Next-Generation Sequencing: Expression Levels of miR-126-3p and piR-5936 Increase with Higher Histologic Grades

Alexandru A. Sabo, Giovanni Birolo, Alessio Naccarati, Mihnea P. Dragomir, Serena Aneli, Alessandra Allione, Marco Oderda and Marco Allasia, Paolo Gontero, Carlotta Sacerdote, Paolo Vineis, Giuseppe Matullo, Barbara Pardini

Table S1. Summary of sequencing results.

Subjects	Specimen	Raw Reads	Clean Reads	Mapped Reads	Assigned Reads	Clean Reads (%)	Mapped Reads (%)	Assigned Reads (%)
BLAP1420	Plasma EVs	17671954	12055669	5223483	1666816	68.2%	29.6%	9.4%
BLAP1449	Plasma EVs	16633993	10451436	3692362	740810	62.8%	22.2%	4.5%
BLAP1455	Plasma EVs	26689776	16930176	5790562	625511	63.4%	21.7%	2.3%
BLAP1511	Plasma EVs	22602738	14810961	5022031	501872	65.5%	22.2%	2.2%
BLAP1520	Plasma EVs	24594736	15338664	4832380	408004	62.4%	19.6%	1.7%
BLAP1530	Plasma EVs	25493380	16296965	6221338	1653829	63.9%	24.4%	6.5%
BLAP1543	Plasma EVs	30644548	20267302	6883415	690186	66.1%	22.5%	2.3%
BLAP1553	Plasma EVs	22494957	13648154	4337710	398214	60.7%	19.3%	1.8%
BLAP1562	Plasma EVs	37097023	22494239	6980918	719338	60.6%	18.8%	1.9%
BLAP1568	Plasma EVs	14005305	8872204	2941084	354375	63.3%	21.0%	2.5%
BLAP1586	Plasma EVs	23277707	14719719	4517336	293844	63.2%	19.4%	1.3%
BLAP1593	Plasma EVs	24375694	15179796	4840539	459439	62.3%	19.9%	1.9%
BLAP1597	Plasma EVs	13306613	11468098	5271827	833274	86.2%	39.6%	6.3%
BLAP1615	Plasma EVs	13625460	11964750	4622927	464958	87.8%	33.9%	3.4%
BLAP1626	Plasma EVs	8368783	7506752	4163757	1896699	89.7%	49.8%	22.7%
BLAP1639	Plasma EVs	9717230	8366821	3233221	324746	86.1%	33.3%	3.3%
BLAP1650	Plasma EVs	7973469	6878670	3037625	777355	86.3%	38.1%	9.7%
BLAP1661	Plasma EVs	6537751	5660168	2349132	378124	86.6%	35.9%	5.8%
BLAP1672	Plasma EVs	6575689	5615703	2574603	875774	85.4%	39.2%	13.3%
BLAP1686	Plasma EVs	6652250	5893398	2904056	923763	88.6%	43.7%	13.9%

BLAP1695	Plasma EVs	6049233	5314007	2238319	433854	87.8%	37.0%	7.2%
BLAP1698	Plasma EVs	8859235	7747248	3047201	517346	87.4%	34.4%	5.8%
BLAP1699	Plasma EVs	10794717	9179345	3470672	491836	85.0%	32.2%	4.6%
BLAP1706	Plasma EVs	15129296	12886390	5133838	990898	85.2%	33.9%	6.5%
BLAP1415	Plasma EVs	12009	9323	3785	278	77.6%	31.5%	2.3%
BLAP1429	Plasma EVs	5973794	4294298	1217928	149363	71.9%	20.4%	2.5%
BLAP1432	Plasma EVs	8183368	6066816	1882353	371281	74.1%	23.0%	4.5%
BLAP1441	Plasma EVs	3517757	2483366	670617	44196	70.6%	19.1%	1.3%
BLAP1443	Plasma EVs	12601615	9765109	3921949	1684608	77.5%	31.1%	13.4%
BLAP1458	Plasma EVs	4578156	3169317	887074	108567	69.2%	19.4%	2.4%
BLAP1488	Plasma EVs	10859436	8276504	3052391	1057142	76.2%	28.1%	9.7%
BLAP1548	Plasma EVs	5546523	3953781	1077451	81031	71.3%	19.4%	1.5%
BLAP1573	Plasma EVs	7598602	5513171	1584187	186398	72.6%	20.8%	2.5%
BLAP1574	Plasma EVs	9799177	7169782	2045536	228321	73.2%	20.9%	2.3%
BLAP1575	Plasma EVs	5651966	4033307	1088389	91801	71.4%	19.3%	1.6%
BLAP1587	Plasma EVs	3546293	2615341	724952	93747	73.7%	20.4%	2.6%
BLAP1608	Plasma EVs	5013906	3682756	1193521	255682	73.5%	23.8%	5.1%
BLAP1616	Plasma EVs	7786438	5929036	1906740	311769	76.1%	24.5%	4.0%
BLAP1619	Plasma EVs	5031097	3685168	1136504	137385	73.2%	22.6%	2.7%
BLAP1625	Plasma EVs	7514601	6011370	2740238	1175133	80.0%	36.5%	15.6%
BLAP1651	Plasma EVs	6260933	4587000	1392849	170071	73.3%	22.2%	2.7%
BLAP1658	Plasma EVs	10504505	8731530	5221718	2336452	83.1%	49.7%	22.2%
BLAP1665	Plasma EVs	7433698	5157131	1614450	333447	69.4%	21.7%	4.5%
BLAP1666	Plasma EVs	9734967	7396568	2392433	471621	76.0%	24.6%	4.8%
BLAP1674	Plasma EVs	2602034	1817754	519605	56606	69.9%	20.0%	2.2%
BLAP1685	Plasma EVs	2726756	1933538	549219	45925	70.9%	20.1%	1.7%
BLAP1703	Plasma EVs	7188579	4712934	1265222	99811	65.6%	17.6%	1.4%
BLAP1704	Plasma EVs	2873862	1992975	790142	333471	69.3%	27.5%	11.6%
BLAP1416	Plasma EVs	8686449	5185701	2717583	1064450	59.7%	31.3%	12.3%
BLAP1418	Plasma EVs	14628177	9894432	4712900	1627630	67.6%	32.2%	11.1%
BLAP1427	Plasma EVs	13188067	7203342	4587363	2909935	54.6%	34.8%	22.1%

BLAP1450	Plasma EVs	17683531	12266347	8529722	3313379	69.4%	48.2%	18.7%
BLAP1460	Plasma EVs	34156281	22273505	13653621	7084645	65.2%	40.0%	20.7%
BLAP1487	Plasma EVs	13994110	7261647	3013566	586035	51.9%	21.5%	4.2%
BLAP1493	Plasma EVs	21650832	17389159	8402978	3352480	80.3%	38.8%	15.5%
BLAP1495	Plasma EVs	43870573	20862407	5137916	2489919	47.6%	11.7%	5.7%
BLAP1497	Plasma EVs	11988977	7942749	3869500	1179577	66.3%	32.3%	9.8%
BLAP1531	Plasma EVs	15983255	11393211	6874627	3431146	71.3%	43.0%	21.5%
BLAP1549	Plasma EVs	28865046	20151959	12811801	6638936	69.8%	44.4%	23.0%
BLAP1556	Plasma EVs	23619841	16138537	10286747	5080941	68.3%	43.6%	21.5%
BLAP1560	Plasma EVs	7233767	5357759	2158164	420657	74.1%	29.8%	5.8%
BLAP1570	Plasma EVs	6216493	4902699	2032883	497538	78.9%	32.7%	8.0%
BLAP1572	Plasma EVs	9651261	7804240	4550120	1880941	80.9%	47.1%	19.5%
BLAP1581	Plasma EVs	8865691	7681757	5390947	2770688	86.6%	60.8%	31.3%
BLAP1590	Plasma EVs	5321844	4292815	2585521	1192487	80.7%	48.6%	22.4%
BLAP1598	Plasma EVs	13081744	9364768	4707703	1668406	71.6%	36.0%	12.8%
BLAP1599	Plasma EVs	10820895	8764650	4802802	1967863	81.0%	44.4%	18.2%
BLAP1604	Plasma EVs	16924800	12072680	5166984	1621993	71.3%	30.5%	9.6%
BLAP1627	Plasma EVs	11240231	9810937	6721830	3201420	87.3%	59.8%	28.5%
BLAP1644	Plasma EVs	19024508	16833689	12892531	7147648	88.5%	67.8%	37.6%
BLAP1662	Plasma EVs	10062688	8100016	4222008	1193398	80.5%	42.0%	11.9%
BLAP1679	Plasma EVs	15201371	11916420	5618404	2455500	78.4%	37.0%	16.2%
BLAP1433	Plasma EVs	3306677	3204448	2004206	879175	96.9%	60.6%	26.6%
BLAP1436	Plasma EVs	13902112	13375931	7204990	2490692	96.2%	51.8%	17.9%
BLAP1452	Plasma EVs	14144414	13590915	6532356	1228480	96.1%	46.2%	8.7%
BLAP1461	Plasma EVs	18695113	18156562	11686382	5663625	97.1%	62.5%	30.3%
BLAP1462	Plasma EVs	13218739	12786593	6749624	1978901	96.7%	51.1%	15.0%
BLAP1463	Plasma EVs	11065478	10611189	5545406	1524471	95.9%	50.1%	13.8%
BLAP1486	Plasma EVs	5976330	5729576	2580410	423873	95.9%	43.2%	7.1%
BLAP1492	Plasma EVs	7483083	7105609	3115503	384240	95.0%	41.6%	5.1%
BLAP1521	Plasma EVs	8678526	8176076	3490919	332673	94.2%	40.2%	3.8%
BLAP1524	Plasma EVs	16040707	15263065	7968625	2444824	95.2%	49.7%	15.2%

BLAP1528	Plasma EVs	7100297	6849369	4259373	1952739	96.5%	60.0%	27.5%
BLAP1539	Plasma EVs	8318173	7972821	4499165	572465	95.8%	54.1%	6.9%
BLAP1552	Plasma EVs	7906917	7632917	4444268	843393	96.5%	56.2%	10.7%
BLAP1589	Plasma EVs	5639785	5444529	3015922	362691	96.5%	53.5%	6.4%
BLAP1600	Plasma EVs	5687666	5497409	3244719	639162	96.7%	57.0%	11.2%
BLAP1623	Plasma EVs	6656015	6240709	3347969	339857	93.8%	50.3%	5.1%
BLAP1629	Plasma EVs	9315350	8994801	5603018	1563673	96.6%	60.1%	16.8%
BLAP1633	Plasma EVs	5176762	4964434	2758895	397639	95.9%	53.3%	7.7%
BLAP1634	Plasma EVs	2746717	2602657	1422244	214312	94.8%	51.8%	7.8%
BLAP1673	Plasma EVs	5574012	5244638	2769157	270521	94.1%	49.7%	4.9%
BLAP1675	Plasma EVs	12492873	12205752	9713216	679280	97.7%	77.8%	5.4%
BLAP1693	Plasma EVs	4657952	4420257	2424747	203908	94.9%	52.1%	4.4%
BLAU1420	Urine	1384406	504238	349688	179241	36.4%	25.3%	12.9%
BLAU1449	Urine	715721	564107	441151	256083	78.8%	61.6%	35.8%
BLAU1455	Urine	48877092	44523717	20776412	3179787	91.1%	42.5%	6.5%
BLAU1460	Urine	3220838	1886379	1186941	604054	58.6%	36.9%	18.8%
BLAU1497	Urine	18758392	18479643	17765953	11925623	98.5%	94.7%	63.6%
BLAU1553	Urine	3103004	2385282	1247787	396470	76.9%	40.2%	12.8%
BLAU1562	Urine	2103760	1182890	618687	245300	56.2%	29.4%	11.7%
BLAU1568	Urine	2685500	2067053	1491254	673471	77.0%	55.5%	25.1%
BLAU1586	Urine	5948433	3638824	1022426	42727	61.2%	17.2%	0.7%
BLAU1593	Urine	1484603	769411	568195	327830	51.8%	38.3%	22.1%
BLAU1597	Urine	5824353	4264349	3149997	1489304	73.2%	54.1%	25.6%
BLAU1615	Urine	9105745	8517595	8296516	5154561	93.5%	91.1%	56.6%
BLAU1626	Urine	3424164	1839248	787864	207103	53.7%	23.0%	6.0%
BLAU1639	Urine	7807668	7290761	6448852	2055099	93.4%	82.6%	26.3%
BLAU1650	Urine	600973	427570	195446	48139	71.1%	32.5%	8.0%
BLAU1661	Urine	189522	156134	72704	11383	82.4%	38.4%	6.0%
BLAU1672	Urine	308277	245267	136037	42348	79.6%	44.1%	13.7%
BLAU1686	Urine	13012648	12355327	10044311	5666517	94.9%	77.2%	43.5%
BLAU1695	Urine	2422413	2145142	1666148	746749	88.6%	68.8%	30.8%

BLAU1698	Urine	6867467	6383728	5825854	3278849	93.0%	84.8%	47.7%
BLAU1415	Urine	5358726	2961543	1885360	1066294	55.3%	35.2%	19.9%
BLAU1416	Urine	12558336	9159567	6180017	2328317	72.9%	49.2%	18.5%
BLAU1418	Urine	31431855	23051080	19556901	11948047	73.3%	62.2%	38.0%
BLAU1427	Urine	1010	536	212	39	53.1%	21.0%	3.9%
BLAU1429	Urine	30446048	25461221	21863790	13669447	83.6%	71.8%	44.9%
BLAU1432	Urine	45308981	23771544	21035111	13642806	52.5%	46.4%	30.1%
BLAU1433	Urine	15476247	7972257	5769780	2961632	51.5%	37.3%	19.1%
BLAU1436	Urine	23205635	16318911	7287021	1683221	70.3%	31.4%	7.3%
BLAU1441	Urine	6400444	3128259	1873081	978296	48.9%	29.3%	15.3%
BLAU1443	Urine	12551001	8087938	6478025	3865714	64.4%	51.6%	30.8%
BLAU1450	Urine	8252538	4590103	3409039	2125895	55.6%	41.3%	25.8%
BLAU1458	Urine	32806571	27452344	23741280	15076448	83.7%	72.4%	46.0%
BLAU1461	Urine	20713763	6559214	4229769	1863294	31.7%	20.4%	9.0%
BLAU1462	Urine	38512377	29541892	26111018	16482368	76.7%	67.8%	42.8%
BLAU1463	Urine	7640225	2847246	1767690	827151	37.3%	23.1%	10.8%
BLAU1487	Urine	8975931	5496628	2759902	1149433	61.2%	30.7%	12.8%
BLAU1488	Urine	24779187	16220021	14180393	9949582	65.5%	57.2%	40.2%
BLAU1492	Urine	5847993	3031911	2198383	1258978	51.8%	37.6%	21.5%
BLAU1552	Urine	7920016	2902101	1922689	1177276	36.6%	24.3%	14.9%
BLAU1570	Urine	8948860	6238527	3367084	1289897	69.7%	37.6%	14.4%
BLAU1572	Urine	13514610	10915173	4788680	1178959	80.8%	35.4%	8.7%
BLAU1573	Urine	7182225	4494081	2574295	1094972	62.6%	35.8%	15.2%
BLAU1599	Urine	22199654	19243366	11981078	3645607	86.7%	54.0%	16.4%
BLAU1618	Urine	12422321	7414437	2340146	167730	59.7%	18.8%	1.4%
BLAU1493	Urine	6162514	1632084	918152	438651	26.5%	14.9%	7.1%
BLAU1556	Urine	19359430	12036471	4509784	861884	62.2%	23.3%	4.5%
BLAU1575	Urine	11932871	7841504	6094560	2955254	65.7%	51.1%	24.8%
BLAU1587	Urine	8214359	3234192	1783494	717555	39.4%	21.7%	8.7%
BLAU1590	Urine	5249868	2958318	2293272	1251013	56.4%	43.7%	23.8%
BLAU1600	Urine	19749597	10576887	7646166	3882186	53.6%	38.7%	19.7%

BLAU1604	Urine	3098327	1143903	410287	72471	36.9%	13.2%	2.3%
BLAU1608	Urine	10676599	6665880	5286232	2902335	62.4%	49.5%	27.2%
BLAU1616	Urine	4691548	2713662	1953748	1083164	57.8%	41.6%	23.1%
BLAU1625	Urine	24966467	19034494	15638420	8764833	76.2%	62.6%	35.1%
BLAU1629	Urine	5816234	1721873	839119	334636	29.6%	14.4%	5.8%
BLAU1633	Urine	10721334	7490036	6529901	3996211	69.9%	60.9%	37.3%
BLAU1634	Urine	10509060	4356312	1809905	634136	41.5%	17.2%	6.0%
BLAU1644	Urine	5595759	4686022	3897696	2233831	83.7%	69.7%	39.9%
BLAU1651	Urine	6047017	4853328	3658635	1982759	80.3%	60.5%	32.8%
BLAU1662	Urine	12628709	10786720	7082582	3621258	85.4%	56.1%	28.7%
BLAU1666	Urine	14718410	9971450	5279250	1904452	67.7%	35.9%	12.9%
BLAU1673	Urine	3692032	2481523	1493996	716487	67.2%	40.5%	19.4%
BLAU1674	Urine	9745877	8123381	7151545	3754734	83.4%	73.4%	38.5%
BLAU1675	Urine	61909253	52757915	31220942	10851322	85.2%	50.4%	17.5%
BLAU1679	Urine	84815741	62924785	19660959	1600459	74.2%	23.2%	1.9%
BLAU1685	Urine	34125602	29151361	22363456	12503660	85.4%	65.5%	36.6%
BLAU1687	Urine	8544808	4870195	2979062	1542554	57.0%	34.9%	18.1%
BLAU1693	Urine	4780334	2491615	1370916	818474	52.1%	28.7%	17.1%
BLAU1452	Urine	4295689	3889333	3687183	2319822	90.5%	85.8%	54.0%
BLAU1486	Urine	1361246	411381	228265	69546	30.2%	16.8%	5.1%
BLAU1495	Urine	1207021	336514	188682	63444	27.9%	15.6%	5.3%
BLAU1511	Urine	1665931	601114	446316	174952	36.1%	26.8%	10.5%
BLAU1520	Urine	1600250	544434	395924	172432	34.0%	24.7%	10.8%
BLAU1521	Urine	4355835	1997047	1726631	1001644	45.8%	39.6%	23.0%
BLAU1524	Urine	2998588	1216844	956878	385853	40.6%	31.9%	12.9%
BLAU1528	Urine	4898718	3847229	3346867	1637462	78.5%	68.3%	33.4%
BLAU1530	Urine	10074874	9382334	9088361	6100845	93.1%	90.2%	60.6%
BLAU1531	Urine	902399	501744	364312	143978	55.6%	40.4%	16.0%
BLAU1532	Urine	7480969	6566350	4393622	1985595	87.8%	58.7%	26.5%
BLAU1539	Urine	8896937	8132361	6738904	2861263	91.4%	75.7%	32.2%
BLAU1543	Urine	11258980	7597113	4973329	2887755	67.5%	44.2%	25.6%

BLAU1548	Urine	24288216	21193861	20683484	13662278	87.3%	85.2%	56.3%
BLAU1549	Urine	6667090	2099304	1709078	1049301	31.5%	25.6%	15.7%
BLAU1560	Urine	3850079	909926	740527	371747	23.6%	19.2%	9.7%
BLAU1579	Urine	7335222	6316602	6160391	3872997	86.1%	84.0%	52.8%
BLAU1581	Urine	8398198	7011998	6605728	4138973	83.5%	78.7%	49.3%
BLAU1589	Urine	1952918	639516	481624	192672	32.7%	24.7%	9.9%
BLAU1598	Urine	8780629	7375930	6954361	4260988	84.0%	79.2%	48.5%
BLAU1623	Urine	7800385	6502409	6217819	3611360	83.4%	79.7%	46.3%
BLAU1627	Urine	2281367	1174744	1032021	589401	51.5%	45.2%	25.8%
BLAU1658	Urine	10783359	9266434	8592999	5236665	85.9%	79.7%	48.6%
BLAU1665	Urine	1777459	1050506	940601	527747	59.1%	52.9%	29.7%
BLAU1417	Urine	2866927	1450547	604501	283823	50.6%	21.1%	9.9%
BLAU1423	Urine	3604066	959414	461672	169044	26.6%	12.8%	4.7%
BLAU1424	Urine	4336266	3181774	2801195	1571214	73.4%	64.6%	36.2%
BLAU1425	Urine	5580899	2313047	1376057	734052	41.4%	24.7%	13.2%
BLAU1445	Urine	3328812	2524371	1673732	640126	75.8%	50.3%	19.2%
BLAU1454	Urine	836606	287254	168277	84204	34.3%	20.1%	10.1%
BLAU1504	Urine	2046644	818511	525016	293418	40.0%	25.7%	14.3%
BLAU1526	Urine	19056280	14782992	9829070	3902412	77.6%	51.6%	20.5%
BLAU1567	Urine	2969985	1906132	1416322	946286	64.2%	47.7%	31.9%
BLAU1574	Urine	5844631	2360284	1422239	695175	40.4%	24.3%	11.9%
BLAU1576	Urine	3291364	2130900	1511903	786727	64.7%	45.9%	23.9%
BLAU1580	Urine	8337736	5644194	4429043	2720748	67.7%	53.1%	32.6%
BLAU1594	Urine	9260974	2297063	1358991	730197	24.8%	14.7%	7.9%
BLAU1617	Urine	3736469	2071461	1403773	772832	55.4%	37.6%	20.7%
BLAU1619	Urine	10370367	5755139	4513441	2366704	55.5%	43.5%	22.8%
BLAU1663	Urine	4549218	1442086	474741	177742	31.7%	10.4%	3.9%
BLAU1668	Urine	15919500	10432374	7108828	2801172	65.5%	44.7%	17.6%
BLAU1677	Urine	4026323	2437350	1387889	597256	60.5%	34.5%	14.8%
BLAU1691	Urine	3325944	2107266	1299393	477113	63.4%	39.1%	14.3%
BLAU1699	Urine	4264090	749855	376882	146292	17.6%	8.8%	3.4%

BLAU1703	Urine	12984684	9053105	7098768	2889192	69.7%	54.7%	22.3%
BLAU1704	Urine	4372645	975026	460734	147594	22.3%	10.5%	3.4%
BLAU1706	Urine	3112126	1312763	673113	241531	42.2%	21.6%	7.8%
BLAU1710	Urine	6230680	3475029	2237959	1114418	55.8%	35.9%	17.9%

Legend:

Raw Reads	Number of reads produced by sequencer
Clean Reads	Number of reads after adapter removal by cutadapt
Mapped Reads	Number of reads mapped by bwa
Assigned Reads	Number of reads that were assigned to a sncRNA
Clean Reads (%)	Percentage of clean reads with respect to total raw reads
Mapped Reads (%)	Percentage of mapped reads with respect to total raw reads
Assigned Reads (%)	Percentage of assigned reads with respect to total raw reads

Table S2. Enrichment analyses for the validated target genes of significantly DE miRNAs using the miRWalk 3.0 database and EnrichR: A) miR-126-3p; B) miR-4508; and C) miR-450b. Only significant enrichments are shown.

**A**					
**hsa-miR-126-3p**					
	**Term**	**Overlap**	***p*-Value**	**Adjusted *p*-Value**	**Genes**
KEGG	Endometrial cancer	1/58	0.012	**0.043**	*FOXO3*
	Amyotrophic lateral sclerosis (ALS)	1/51	0.010	**0.043**	*GRIN2B*
	Intestinal immune network for IgA production	1/48	0.010	**0.043**	*CXCL12*
	Cocaine addiction	1/49	0.010	**0.043**	*GRIN2B*
	Rheumatoid arthritis	1/91	0.018	**0.043**	*CXCL12*
	NF-kappa B signaling pathway	1/95	0.019	**0.043**	*CXCL12*
	Prolactin signaling pathway	1/70	0.014	**0.043**	*FOXO3*
	Nicotine addiction	1/40	0.008	**0.043**	*GRIN2B*
	Non-small cell lung cancer	1/66	0.013	**0.043**	*FOXO3*
	Neurotrophin signaling pathway	1/119	0.024	**0.043**	*FOXO3*

	Amphetamine addiction	1/68	0.014	**0.043**	*GRIN2B*
	Mitophagy	1/65	0.013	**0.043**	*FOXO3*
	AMPK signaling pathway	1/120	0.024	**0.043**	*FOXO3*
	Long-term potentiation	1/67	0.013	**0.043**	*GRIN2B*
	Systemic lupus erythematosus	1/133	0.026	**0.043**	*GRIN2B*
	Circadian entrainment	1/97	0.019	**0.043**	*GRIN2B*
	Leukocyte transendothelial migration	1/112	0.022	**0.043**	*CXCL12*
	Longevity regulating pathway	1/102	0.020	**0.043**	*FOXO3*
	Glutamatergic synapse	1/114	0.023	**0.043**	*GRIN2B*
	Chemokine signaling pathway	2/190	0.001	**0.019**	*CXCL12; FOXO3*
	Dopaminergic synapse	1/131	0.026	**0.043**	*GRIN2B*
	FoxO signaling pathway	1/132	0.026	**0.043**	*FOXO3*
	Alzheimer disease	1/171	0.034	**0.049**	*GRIN2B*
	Cellular senescence	1/160	0.032	**0.049**	*FOXO3*
	Huntington disease	1/193	0.038	**0.051**	*GRIN2B*
	Axon guidance	1/181	0.036	**0.049**	*CXCL12*
	Alcoholism	1/180	0.036	**0.049**	*GRIN2B*
**GO Biological Process**	positive regulation of neuron death (GO:1901216)	1/39	0.000	**0.002**	*FOXO3; GRIN2B*
	regulation of dendritic spine maintenance (GO:1902950)	1/8	0.002	**0.016**	*GRIN2B*
	negative regulation of cellular extravasation (GO:0002692)	1/7	0.001	**0.016**	*CXCL12*
	positive regulation of calcium ion import (GO:0090280)	1/11	0.002	**0.016**	*CXCL12*
	excitatory chemical synaptic transmission (GO:0098976)	1/8	0.002	**0.016**	*GRIN2B*
	negative regulation of dendritic cell apoptotic process (GO:2000669)	1/8	0.002	**0.016**	*CXCL12*

regulation of actin filament length (GO:0030832)	1/11	0.002	**0.016**	*CXCL12*
integrin activation (GO:0033622)	1/9	0.002	**0.016**	*CXCL12*
induction of positive chemotaxis (GO:0050930)	1/11	0.002	**0.016**	*CXCL12*
regulation of intrinsic apoptotic signaling pathway in response to DNA damage (GO:1902229)	1/17	0.003	**0.016**	*CXCL12*
regulation of dendritic cell apoptotic process (GO:2000668)	1/11	0.002	**0.016**	*CXCL12*
positive regulation of positive chemotaxis (GO:0050927)	1/17	0.003	**0.016**	*CXCL12*
protein heterotetramerization (GO:0051290)	1/20	0.004	**0.016**	*GRIN2B*
positive regulation of neuron apoptotic process (GO:0043525)	1/20	0.004	**0.016**	*FOXO3*
positive regulation of lymphocyte migration (GO:2000403)	1/18	0.004	**0.016**	*CXCL12*
response to ethanol (GO:0045471)	1/15	0.003	**0.016**	*GRIN2B*
negative regulation of leukocyte apoptotic process (GO:2000107)	1/13	0.003	**0.016**	*CXCL12*
regulation of calcium ion import (GO:0090279)	1/23	0.005	**0.016**	*CXCL12*
mitochondrial RNA metabolic process (GO:0000959)	1/21	0.004	**0.016**	*FOXO3*
positive regulation of monocyte chemotaxis (GO:0090026)	1/18	0.004	**0.016**	*CXCL12*
regulation of monocyte chemotaxis (GO:0090025)	1/23	0.005	**0.016**	*CXCL12*
positive regulation of T cell migration (GO:2000406)	1/21	0.004	**0.016**	*CXCL12*
excitatory postsynaptic potential (GO:0060079)	1/20	0.004	**0.016**	*GRIN2B*
transcription from mitochondrial promoter (GO:0006390)	1/13	0.003	**0.016**	*FOXO3*

positive regulation of endopeptidase activity (GO:0010950)	1/25	0.005	**0.017**	*GRIN2B*
negative regulation of intrinsic apoptotic signaling pathway in response to DNA damage (GO:1902230)	1/25	0.005	**0.017**	*CXCL12*
calcium ion transport into cytosol (GO:0060402)	1/20	0.004	**0.016**	*GRIN2B*
regulation of cysteine-type endopeptidase activity (GO:2000116)	1/37	0.007	**0.017**	*GRIN2B*
positive regulation of mononuclear cell migration (GO:0071677)	1/20	0.004	**0.016**	*CXCL12*
positive regulation of erythrocyte differentiation (GO:0045648)	1/23	0.005	**0.016**	*FOXO3*
regulation of actin polymerization or depolymerization (GO:0008064)	1/29	0.006	**0.017**	*CXCL12*
positive regulation of calcium ion transport (GO:0051928)	1/34	0.007	**0.017**	*CXCL12*
response to alcohol (GO:0097305)	1/26	0.005	**0.017**	*GRIN2B*
glutamate receptor signaling pathway (GO:0007215)	1/37	0.007	**0.017**	*GRIN2B*
regulation of T cell migration (GO:2000404)	1/18	0.004	**0.016**	*CXCL12*
long-term synaptic potentiation (GO:0060291)	1/22	0.004	**0.016**	*GRIN2B*
protein kinase B signaling (GO:0043491)	1/36	0.007	**0.017**	*FOXO3*
posttranscriptional regulation of gene expression (GO:0010608)	1/45	0.009	**0.019**	*FOXO3*
negative regulation of response to DNA damage stimulus (GO:2001021)	1/29	0.006	**0.017**	*CXCL12*
regulation of supramolecular fiber organization (GO:1902903)	1/27	0.005	**0.017**	*CXCL12*

positive regulation of myeloid cell differentiation (GO:0045639)	1/30	0.006	**0.017**	*FOXO3*
positive regulation of cell death (GO:0010942)	1/58	0.012	**0.022**	*GRIN2B*
blood circulation (GO:0008015)	1/51	0.010	**0.020**	*CXCL12*
protein heterooligomerization (GO:0051291)	1/50	0.010	**0.020**	*GRIN2B*
calcium ion transmembrane import into cytosol (GO:0097553)	1/37	0.007	**0.017**	*GRIN2B*
regulation of cellular protein metabolic process (GO:0032268)	1/51	0.010	**0.020**	*FOXO3*
positive regulation of cell adhesion (GO:0045785)	1/65	0.013	**0.023**	*CXCL12*
protein tetramerization (GO:0051262)	1/91	0.018	**0.028**	*GRIN2B*
positive regulation of homeostatic process (GO:0032846)	1/84	0.017	**0.028**	*FOXO3*
positive regulation of cysteine-type endopeptidase activity (GO:2001056)	1/56	0.011	**0.021**	*GRIN2B*
regulation of cellular amide metabolic process (GO:0034248)	1/39	0.008	**0.018**	*FOXO3*
regulation of postsynaptic membrane potential (GO:0060078)	1/34	0.007	**0.017**	*GRIN2B*
cellular metal ion homeostasis (GO:0006875)	35431	0.019	**0.029**	*CXCL12*
cellular response to oxidative stress (GO:0034599)	1/116	0.023	**0.034**	*FOXO3*
negative regulation of cell projection organization (GO:0031345)	1/42	0.008	**0.019**	*GRIN2B*
chemical synaptic transmission, postsynaptic (GO:0099565)	1/34	0.007	**0.017**	*GRIN2B*
regulation of erythrocyte differentiation (GO:0045646)	1/33	0.007	**0.017**	*FOXO3*

cytokine-mediated signaling pathway (GO:0019221)	2/634	0.006	**0.017**	*CXCL12; FOXO3*
mitochondrial gene expression (GO:0140053)	1/46	0.009	**0.019**	*FOXO3*
positive regulation of leukocyte chemotaxis (GO:0002690)	1/62	0.012	**0.022**	*CXCL12*
cellular divalent inorganic cation homeostasis (GO:0072503)	1/102	0.020	**0.031**	*CXCL12*
cell chemotaxis (GO:0060326)	1/61	0.012	**0.022**	*CXCL12*
regulation of neuron death (GO:1901214)	1/73	0.015	**0.025**	*GRIN2B*
chemokine-mediated signaling pathway (GO:0070098)	1/53	0.011	**0.021**	*CXCL12*
positive regulation of synaptic transmission (GO:0050806)	1/68	0.014	**0.023**	*GRIN2B*
protein complex assembly (GO:0006461)	1/190	0.037	**0.051**	*CXCL12*
negative regulation of intrinsic apoptotic signaling pathway (GO:2001243)	1/63	0.013	**0.022**	*CXCL12*
calcium ion homeostasis (GO:0055074)	1/88	0.017	**0.028**	*CXCL12*
ephrin receptor signaling pathway (GO:0048013)	1/83	0.016	**0.028**	*GRIN2B*
axon guidance (GO:0007411)	1/159	0.031	**0.044**	*CXCL12*
calcium ion transmembrane transport (GO:0070588)	1/87	0.017	**0.028**	*GRIN2B*
regulation of neuron apoptotic process (GO:0043523)	1/90	0.018	**0.028**	*FOXO3*
tumor necrosis factor-mediated signaling pathway (GO:0033209)	1/124	0.025	**0.035**	*FOXO3*
brain development (GO:0007420)	1/120	0.024	**0.035**	*GRIN2B*
cellular calcium ion homeostasis (GO:0006874)	1/124	0.025	**0.035**	*CXCL12*

	Term	Overlap	*p*-Value	Adjusted *p*-Value	Genes
	positive regulation of cell motility (GO:2000147)	1/180	0.036	**0.049**	*CXCL12*
GO Molecular Functions	NMDA glutamate receptor activity (GO:0004972)	1/8	0.002	**0.017**	*GRIN2B*
	CXCR chemokine receptor binding (GO:0045236)	1/18	0.004	**0.017**	*CXCL12*
	glycine binding (GO:0016594)	1/14	0.003	**0.017**	*GRIN2B*
	voltage-gated ion channel activity (GO:0005244)	1/22	0.004	**0.017**	*GRIN2B*
	ionotropic glutamate receptor activity (GO:0004970)	1/17	0.003	**0.017**	*GRIN2B*
	ligand-gated calcium channel activity (GO:0099604)	1/21	0.004	**0.017**	*GRIN2B*
	amino acid binding (GO:0016597)	1/40	0.008	**0.025**	*GRIN2B*
	chemokine activity (GO:0008009)	1/47	0.009	**0.025**	*CXCL12*
	chemokine receptor binding (GO:0042379)	1/50	0.010	**0.025**	*CXCL12*
	G-protein coupled receptor binding (GO:0001664)	1/134	0.027	**0.045**	*CXCL12*
	cytokine receptor binding (GO:0005126)	1/138	0.027	**0.045**	*CXCL12*
	ligand-gated cation channel activity (GO:0099094)	1/61	0.012	**0.028**	*GRIN2B*
	transcriptional repressor activity, RNA polymerase II transcription regulatory region sequence-specific binding (GO:0001227)	1/160	0.032	**0.045**	*FOXO3*
	integrin binding (GO:0005178)	1/95	0.019	**0.037**	*CXCL12*
	core promoter binding (GO:0001047)	1/98	0.019	**0.037**	*FOXO3*
	cytokine activity (GO:0005125)	1/156	0.031	**0.045**	*CXCL12*

B

hsa-miR-4508

	**Term**	**Overlap**	***p*-Value**	**Adjusted *p*-Value**	**Genes**
GO ¦	Fc-gamma receptor signaling pathway (GO:0038094)	3/135	0.000	**0.005**	*VAV3; YES1; ABL1*

	peptidyl-tyrosine autophosphorylation (GO:0038083)	2/46	0.000	**0.021**	*YES1; ABL1*
	Fc-gamma receptor signaling pathway involved in phagocytosis (GO:0038096)	3/134	0.000	**0.005**	*VAV3; YES1; ABL1*
	Fc receptor mediated stimulatory signaling pathway (GO:0002431)	3/136	0.000	**0.005**	*VAV3; YES1; ABL1*
	transmembrane receptor protein tyrosine kinase signaling pathway (GO:0007169)	3/397	0.001	**0.038**	*VAV3; YES1; ABL1*
	negative regulation of protein ubiquitination (GO:0031397)	2/51	0.000	**0.021**	*U2AF2; ABL1*
	enzyme linked receptor protein signaling pathway (GO:0007167)	2/121	0.002	**0.048**	*YES1; ABL1*
	cellular protein modification process (GO:0006464)	4/1002	0.002	**0.048**	*YES1; ABL1; ASB6; IRAK3*
	negative regulation of protein serine/threonine kinase activity (GO:0071901)	2/68	0.001	**0.031**	*ABL1; IRAK3*
	peptidyl-tyrosine phosphorylation (GO:0018108)	2/80	0.001	**0.032**	*YES1; ABL1*
	protein modification process (GO:0036211)	2/85	0.001	**0.032**	*YES1; ABL1*
	ephrin receptor signaling pathway (GO:0048013)	2/83	0.001	**0.032**	*VAV3; YES1*
**GO Molecular Functions**	protein phosphorylated amino acid binding (GO:0045309)	2/35	0.000	**0.004**	*YES1; ABL1*
	phosphotyrosine residue binding (GO:0001784)	2/30	0.000	**0.004**	*YES1; ABL1*
	pre-mRNA 3'-splice site binding (GO:0030628)	1/09	0.005	**0.035**	*U2AF2*
	non-membrane spanning protein tyrosine kinase activity (GO:0004715)	2/44	0.000	**0.005**	*YES1; ABL1*
	poly-pyrimidine tract binding (GO:0008187)	1/21	0.013	**0.035**	*U2AF2*
	neuropilin binding (GO:0038191)	1/20	0.012	**0.035**	*ABL1*
	magnesium ion binding (GO:0000287)	2/128	0.003	**0.026**	*ABL1; IRAK3*

	Term	Overlap	*p*-Value	Adjusted *p*-Value	Genes
	ephrin receptor binding (GO:0046875)	1/20	0.012	**0.035**	*ABL1*
	GTPase activator activity (GO:0005096)	2/250	0.009	**0.035**	*VAV3; RGS6*
	adenylyltransferase activity (GO:0070566)	1/22	0.013	**0.035**	*ABL1*
	manganese ion binding (GO:0030145)	1/36	0.021	**0.046**	*ABL1*
	calcium-dependent cysteine-type endopeptidase activity (GO:0004198)	1/22	0.013	**0.035**	*CAPN15*
	ATP binding (GO:0005524)	2/256	0.010	**0.035**	*ABL1; IRAK3*
	SH2 domain binding (GO:0042169)	1/27	0.016	**0.038**	*ABL1*
	GTPase regulator activity (GO:0030695)	2/276	0.011	**0.035**	*VAV3; RGS6*
	pre-mRNA binding (GO:0036002)	1/31	0.018	**0.042**	*U2AF2*
	protein tyrosine kinase activity (GO:0004713)	2/148	0.003	**0.026**	*YES1; ABL1*
	actin monomer binding (GO:0003785)	1/26	0.015	**0.038**	*ABL1*
	protein kinase C binding (GO:0005080)	1/41	0.024	**0.048**	*ABL1*
	protein kinase activity (GO:0004672)	3/514	0.003	**0.026**	*YES1; ABL1; IRAK3*
	purine ribonucleoside triphosphate binding (GO:0035639)	2/397	0.023	**0.047**	*ABL1; IRAK3*
	adenyl ribonucleotide binding (GO:0032559)	2/280	0.012	**0.035**	*ABL1; IRAK3*
	mitogen-activated protein kinase binding (GO:0051019)	1/21	0.013	**0.035**	*ABL1*
**C**					
**hsa-miR-450b-5p**					
	**Term**	**Overlap**	***p*-Value**	**Adjusted *p*-Value**	**Genes**
**KEGG**	Natural killer cell mediated cytotoxicity	1/131	0.0323	**0.032327**	*RAET1E*
**GO Molecular**	alpha-N-acetylneuraminate alpha-2.8-sialyltransferase activity (GO:0003828)	1/7	0.002	**0.004**	*ST8SIA3*
	natural killer cell lectin-like receptor binding (GO:0046703)	1/10	0.002	**0.004**	*RAET1E*

	sialyltransferase activity (GO:0008373)	1/22	0.005	**0.005**	*ST8SIA3*
GO Biological Process	positive regulation of triglyceride biosynthetic process (GO:0010867)	1/10	0.002	**0.013**	*CNEP1R1*
	vacuolar proton-transporting V-type ATPase complex assembly (GO:0070072)	1/10	0.002	**0.013**	*VMA21*
	ganglioside biosynthetic process (GO:0001574)	1/11	0.003	**0.013**	*ST8SIA3*
	positive regulation of triglyceride metabolic process (GO:0090208)	1/17	0.004	**0.013**	*CNEP1R1*
	proton-transporting V-type ATPase complex assembly (GO:0070070)	1/10	0.002	**0.013**	*VMA21*
	regulation of triglyceride biosynthetic process (GO:0010866)	1/14	0.003	**0.013**	*CNEP1R1*
	N-glycan processing (GO:0006491)	1/18	0.004	**0.013**	*ST8SIA3*
	positive regulation of natural killer cell mediated cytotoxicity (GO:0045954)	1/18	0.004	**0.013**	*RAET1E*
	glycosphingolipid biosynthetic process (GO:0006688)	1/18	0.004	**0.013**	*ST8SIA3*
	positive regulation of natural killer cell mediated immunity (GO:0002717)	1/18	0.004	**0.013**	*RAET1E*
	regulation of natural killer cell mediated cytotoxicity (GO:0042269)	1/23	0.006	**0.016**	*RAET1E*
	ganglioside metabolic process (GO:0001573)	1/16	0.004	**0.013**	*ST8SIA3*
	glycosphingolipid metabolic process (GO:0006687)	1/55	0.014	**0.020**	*ST8SIA3*
	positive regulation of leukocyte mediated cytotoxicity (GO:0001912)	1/26	0.006	**0.016**	*RAET1E*
	sialylation (GO:0097503)	1/17	0.004	**0.013**	*ST8SIA3*
	nuclear envelope disassembly (GO:0051081)	1/47	0.012	**0.018**	*CNEP1R1*
	ceramide biosynthetic process (GO:0046513)	1/33	0.008	**0.016**	*ST8SIA3*

regulation of protein dephosphorylation (GO:0035304)	1/41	0.010	**0.018**	*CNEP1R1*
positive regulation of protein dephosphorylation (GO:0035307)	1/32	0.008	**0.016**	*CNEP1R1*
positive regulation of lipid biosynthetic process (GO:0046889)	1/29	0.007	**0.016**	*CNEP1R1*
glycoprotein metabolic process (GO:0009100)	1/49	0.012	**0.018**	*ST8SIA3*
positive regulation of dephosphorylation (GO:0035306)	1/25	0.006	**0.016**	*CNEP1R1*
glycolipid biosynthetic process (GO:0009247)	1/43	0.011	**0.018**	*ST8SIA3*
glycoprotein biosynthetic process (GO:0009101)	1/73	0.018	**0.023**	*ST8SIA3*
mitotic nuclear envelope disassembly (GO:0007077)	1/45	0.011	**0.018**	*CNEP1R1*
protein localization to organelle (GO:0033365)	1/98	0.024	**0.028**	*CNEP1R1*
regulation of ATPase activity (GO:0043462)	1/41	0.010	**0.018**	*VMA21*
positive regulation of cellular biosynthetic process (GO:0031328)	1/160	0.039	**0.043**	*CNEP1R1*
protein localization to nucleus (GO:0034504)	1/97	0.024	**0.028**	*CNEP1R1*
oligosaccharide metabolic process (GO:0009311)	1/64	0.016	**0.022**	*ST8SIA3*
protein glycosylation (GO:0006486)	1/131	0.032	**0.036**	*ST8SIA3*
regulation of hydrolase activity (GO:0051336)	1/81	0.020	**0.025**	*VMA21*
sphingolipid biosynthetic process (GO:0030148)	1/67	0.017	**0.022**	*ST8SIA3*
positive regulation of protein modification process (GO:0031401)	1/164	0.040	**0.043**	*CNEP1R1*

D

hsa-miR-185-5p

Term	Overlap	*p*-Value	Adj *p*-Value	Genes

**WikiPathways**	Regulation of Microtubule Cytoskeleton WP2038	5/46	0.00001	**0.003**	*CDC42;DIAPH1;TAOK1;CAMK4;EPHB2*
**GO Biological Process**	positive regulation of cell cycle arrest (GO:0071158)	5/82	0.0007	**0.001**	*BTG2; ZNF385A; HMGA2; TFDP1; GATA6*
	mitotic DNA damage checkpoint (GO:0044773)	5/86	0.0009	**0.001**	*BTG2; ZNF385A; HMGA2; TFDP1;TAOK1*
	mitotic DNA integrity checkpoint (GO:0044774)	5/90	0.001	**0.001**	*BTG2; ZNF385A; HMGA2; TFDP1;TAOK1*

The enrichment for this class has been done directly with GSEA from miRWalk on GO Biological Processes.

Table S3. Machine learning methods to evaluate the predictivity of sncRNAs in the distinction of bladder cancer: logistic regression with lasso and elastic net penalty, random forest classifier and AdaBoost classifier.

Classification	AdaBoost	ElasticNet C = 0.1	ElasticNet C = 1	Lasso C = 0.1	Lasso C = 1	Random Forest
**BC vs CO plasma**	50.0%	48.0%	55.9%	49.1%	53.7%	51.6%
**BC vs CO urine**	75.5%	77.1%	79.2%	76.4%	76.5%	72.8%
**G1+G2 vs CO plasma**	50.3%	50.0%	48.0%	50.0%	46.4%	50.4%
**G1+G2 vs CO urine**	71.6%	65.8%	74.9%	64.2%	71.5%	68.5%
**G3 vs CO plasma**	48.5%	50.0%	50.0%	50.0%	51.2%	50.0%
**G3 vs CO urine**	79.9%	50.0%	80.8%	50.0%	79.4%	76.0%
**G3 vs G1+G2 plasma**	45.2%	50.0%	49.2%	50.0%	49.2%	46.4%
**G3 vs G1+G2 urine**	56.4%	50.0%	59.5%	50.0%	58.9%	65.5%
**MIBC vs CO plasma**	53.4%	50.0%	49.5%	50.0%	48.9%	50.0%
**MIBC vs CO urine**	72.4%	50.0%	83.0%	50.0%	78.0%	80.0%
**MIBC vs NMIBC plasma**	63.6%	50.0%	61.2%	50.0%	59.9%	50.0%
**MIBC vs NMIBC urine**	57.6%	50.0%	64.1%	50.0%	62.3%	54.1%
**NMIBC vs CO plasma**	51.8%	50.0%	58.5%	50.0%	55.9%	52.8%
**NMIBC vs CO urine**	74.2%	77.4%	74.7%	75.4%	73.2%	72.2%

BC = bladder cancer; CO = controls; MIBC = muscle invasive bladder cancer; NMIBC = non-muscle invasive bladder cancer; G = grade

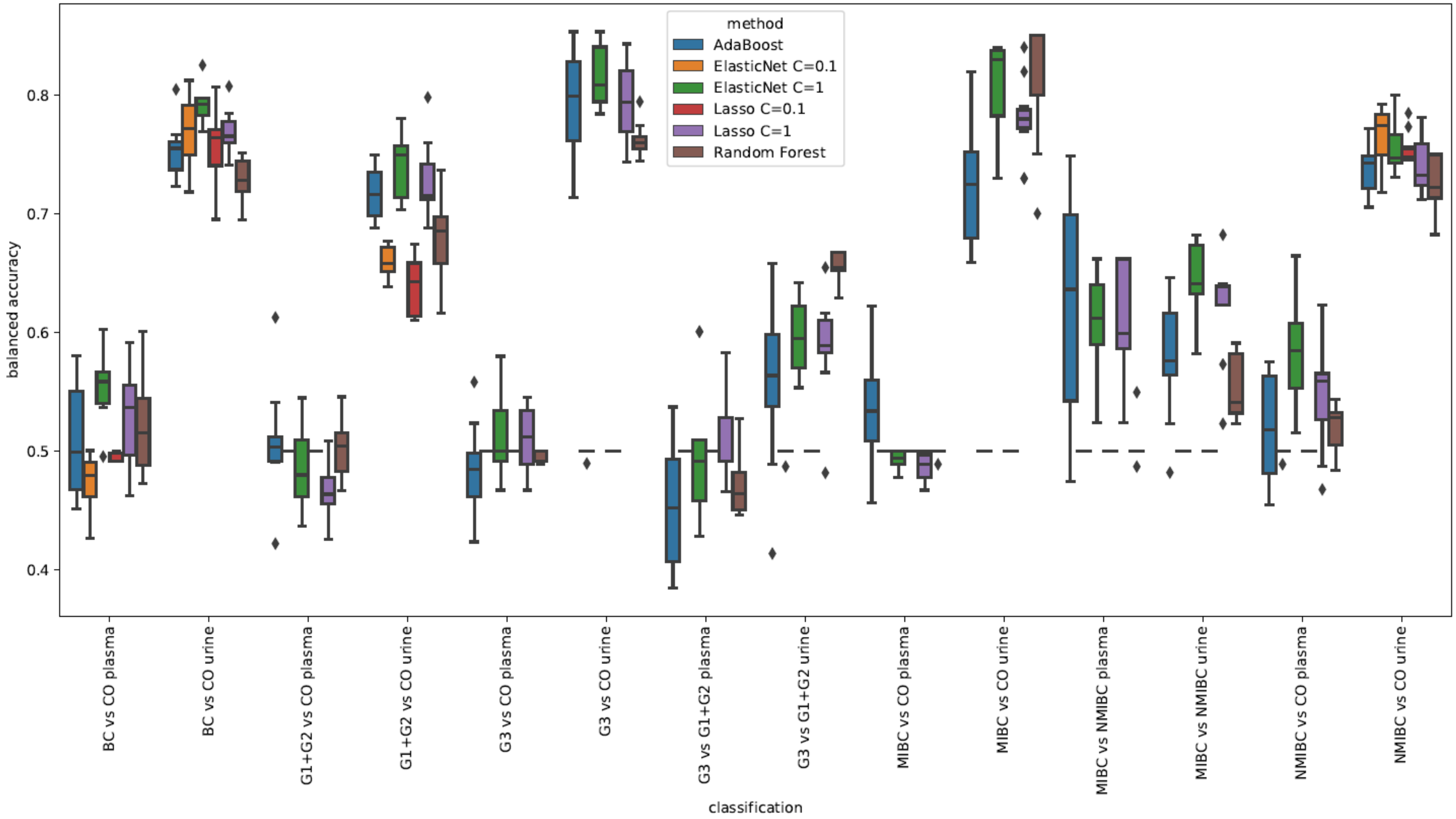

Figure S1. Assessment of the predictivity of sncRNAs from plasma EVs and from urine using different machine learning methods (logistic regression with lasso and elastic net penalty, random forest classifier, and AdaBoost classifier) for some binary classification problems: cases versus controls and pairwise comparisons by tumor type (MIBC, NMIBC and controls) and WHO 1973 histological grade (G3, G1+G2 and controls). Balanced accuracy of 0.5 is the baseline level of a random classifier. In general, urine sncRNAs perform better than their counterparts in EVs.

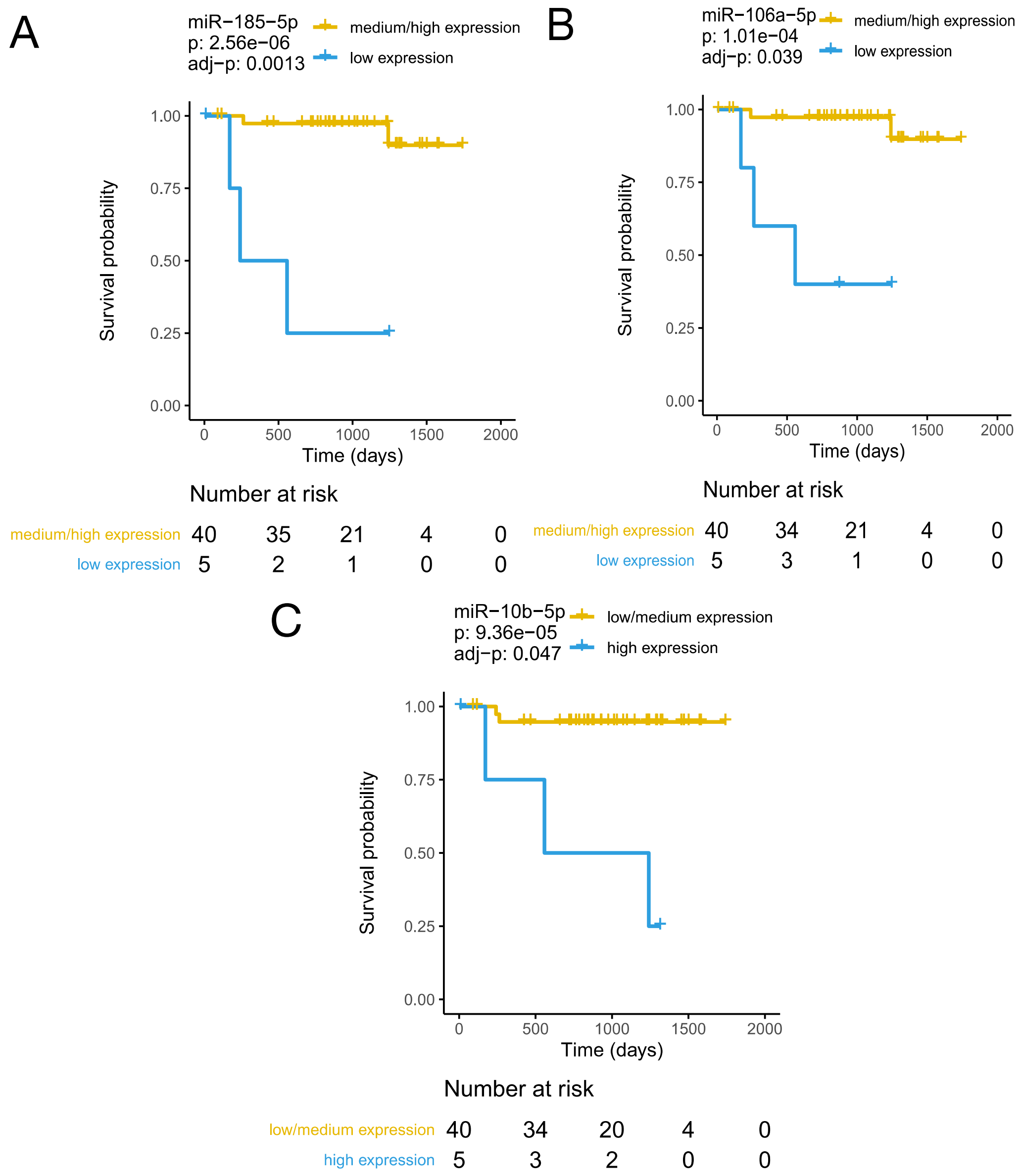

Figure S2. Kaplan–Meier CSM curves in BC patients stratified for the expression levels of: **A**) miR-185-5p; **B**) miR-106a-5p; and **C**) miR-10b-5p.

© 2020 by the authors. Licensee MDPI, Basel, Switzerland. This article is an open access article distributed under the terms and conditions of the Creative Commons Attribution (CC BY) license (http://creativecommons.org/licenses/by/4.0/).
